# Complicated enterocele: timely resolution with bowel resection via a vaginal approach: case report

**DOI:** 10.3389/fsurg.2023.1228981

**Published:** 2023-07-13

**Authors:** Paola Abis, Clelia Madeddu, Giovanni Magro Malosso, Gabriele Sole, Alessia Mereu, Giorgia Locci, Antonio Macciò

**Affiliations:** ^1^ Department of Obstetrics and Gynecology, ARNAS G. Brotzu, Cagliari, Italy; ^2^ Department of Medical Sciences and Public Health, University of Cagliari, Cagliari, Italy; ^3^ Unit of Anatomic Pathology, ARNAS G. Brotzu, Cagliari, Italy; ^4^ Department of Surgical Sciences, University of Cagliari, Cagliari, Italy

**Keywords:** bowel resection, case report, enterocele, evisceration, gynecology, minimally invasive surgery

## Abstract

**Background:**

Enterocele is an uncommon, serious condition that requires accurate and early diagnosis to prevent complications such as intestinal obstruction, incarceration, and strangulation, with consequent intestinal ischemia, necrosis, and evisceration. We report a rare case of a patient with a voluminous enterocele and initial signs of intestinal ischemia who underwent urgent vaginal surgery.

**Case description:**

An 80-year-old woman presented with a voluminous mass protruding from the vagina, associated abdominopelvic pain, a 10-day history of bowel sub-occlusion, and numerous episodes of profuse vaginal bleeding. She was diagnosed with an enterocele with early signs of complications. Owing to her advanced clinical condition and comorbidities, we opted for an urgent vaginal procedure. Intestinal loops with initial signs of ischemia were resected via a transvaginal approach, leading to good clinical outcomes. She was discharged on postoperative day 5.

**Conclusions:**

This rare case highlights a surgical emergency that was managed with transvaginal resection of the intestine. Early identification of the initial signs of complications allowed for this less invasive approach, resulting in reduced morbidity and length of hospital stay.

## 1. Introduction

Intestinal prolapse or enterocele involves herniation of the small intestine into the vaginal canal, creating a bulge. Most enteroceles develop downward between the uterosacral ligaments and the rectovaginal or vesicovaginal space (1). While the precise etiology of abdominal cavity content protrusion into the vagina has not been fully established, increased intra-abdominal pressure is a likely underlying factor. Multiparity, aging, and other processes that place pressure on the pelvic floor can weaken muscle and ligamentous structures supporting the pelvic organs, causing a prolapse of the small bowel (2). The main risk factors for enterocele development are multiparity; previous gynecological surgery, including hysterectomy; old age; obesity; intense physical effort; and paroxysmal episodes of cough, as in patients with asthma or chronic obstructive pulmonary disease (3, 4). Anatomical defects concurrent with enteroceles are essentially fascial defects, such as hernias. The most characteristic types of enterocele are those resulting from prolapse of the vaginal cuff. In particular, the cardinal and uterosacral ligaments suspend the vaginal apex. An apical enterocele occurs when the pubocervical ligament separates from the rectovaginal fascia and when the peritoneum is in contact with the vaginal mucosa, as typically seen after total hysterectomy (5). The organs most prone to prolapse are the distal ileum, omentum, cecal appendix, bladder, and rectum.

A serious but rare enterocele-related complication is evisceration of the enclosed intestinal loops (6), with an associated mortality risk ranging from 6% to 8% (7). Evisceration occurs in the context of severe atrophy of the vaginal mucosa owing to hypoestrogenism (8, 9). Hypoestrogenism, typical in postmenopausal women, produces a significant reduction in the elasticity of the peritoneum, fascia, and vaginal mucosa, leading to subsequent distension and rupture (3). Early intervention is essential as up to 33% of small bowel transvaginal protrusions have been reported to result in intestinal ischemia due to strangulation or subsequent evisceration of the prolapsed loops (10). The first clinical signs of complications often manifest as signs of intestinal occlusion or sub-occlusion; abdominal, pelvic, or vaginal pain; and vaginal bleeding. Identifying early warning signs and symptoms of vaginal rupture with consequent pelvic evisceration is essential for reducing morbidity. Early management requires resolution of the enterocele and gentle reinsertion of the bowel into the peritoneal cavity. Currently, no consensus has been reached in terms of the ideal approach for the surgical management of complications, transabdominal, laparoscopic, or combined management methods have predominantly been reported (11).

Herein, we report a rare case of a patient with a voluminous enterocele and initial signs of vaginal rupture, bowel occlusion, and intestinal ischemia who underwent urgent surgery using a vaginal approach.

## 2. Case description

### 2.1. Patient information

This case report was written in accordance with the CARE guidelines. An 80-year-old overweight woman with hypertension and hypercholesterolemia visited the Oncological Gynecology Department of ARNAS Brotzu of Cagliari, Italy in March 2023, presenting with a voluminous mass protruding from the vagina. Her symptoms included abdominopelvic pain, a 10-day history of sub-occlusion, and multiple episodes of profuse vaginal bleeding. She was also referred for regular but difficult diuresis and bladder tenesmus. These conditions necessitated involuntary bedrest and she was unable to perform standard activities of daily living and self-care. Her gynecological clinical history included three spontaneous vaginal deliveries, an abdominal hysterectomy and bilateral adnexectomy for uterine fibromatosis in 2004, and a vaginal repair of a type III cystocele in 2005. Within a year of her last gynecological surgery, she had a recurrence of a type III genital prolapse but declined to undergo further surgical interventions or gynecological check-ups. Her vaginal cuff prolapse subsequently worsened, leading to the development of an extensive enterocele.

### 2.2. Diagnostic assessment

**Figure 1:**
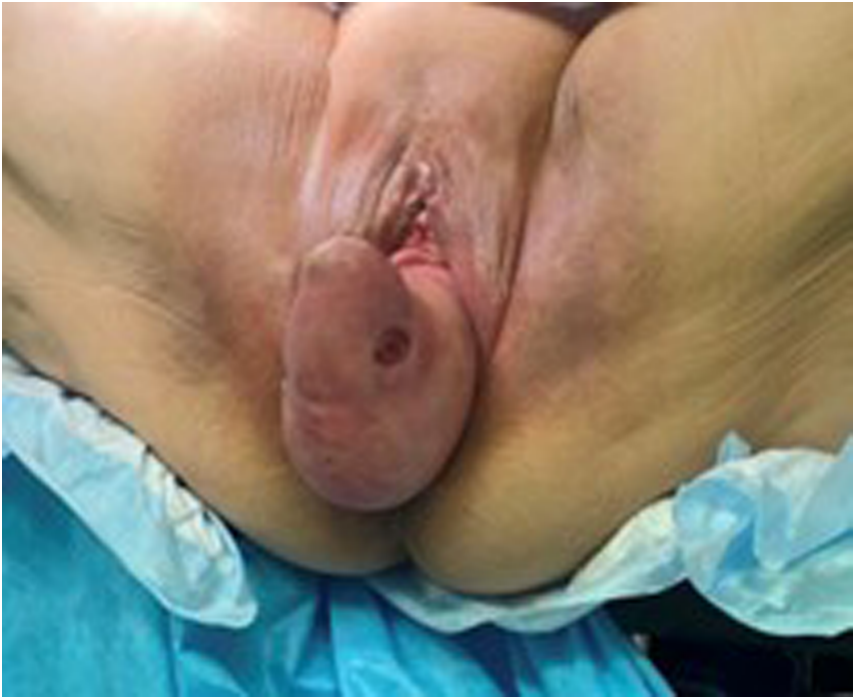
An image of the voluminous mass measuring approximately 15 cm protruding from the vagina showing areas of ulceration.

**Figure 2:**
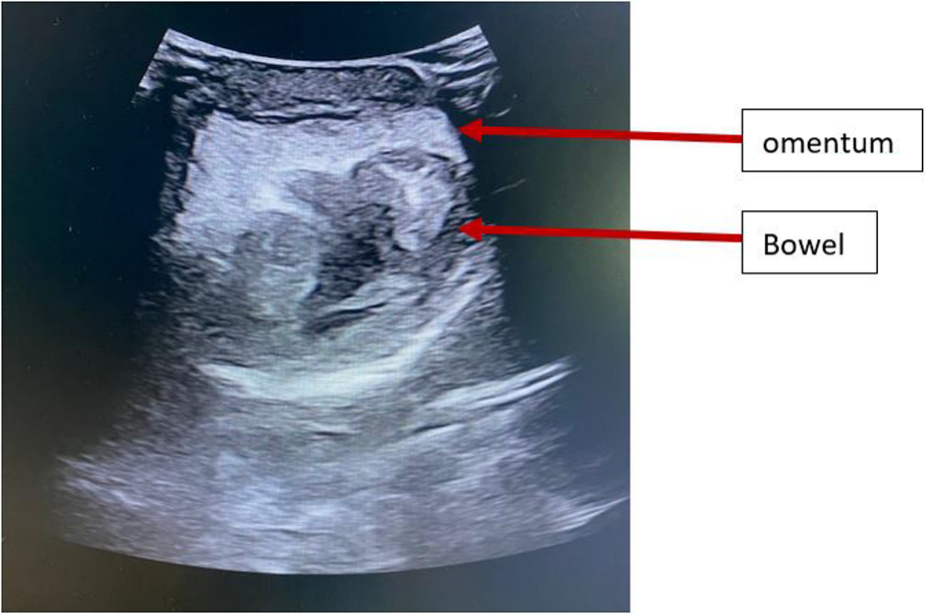
Ultrasonographic image of the vaginal prolapse showing the displaced omentum and bowel loops.

On physical examination, a painful voluminous mass protruded from the vaginal ostium, measuring approximately 15 cm, with a soft consistency, some bleeding ulcerations, and a highly dystrophic area showing initial signs of necrosis in the upper portion (Figure 1). We also noted abdominal distension, pain on deep palpation especially in the lower quadrants, increased enterocolic tympanism, and decreased peristalsis. Abdominal ultrasonography showed a full and normally positioned bladder and a small pelvic effusion. Upon placement of the probe over the vaginal prolapse site, displaced omentum and intestinal loops were visualized, with some having thicker walls (Figure 2). Admission-related laboratory test results showed mild leukocytosis [white blood cells (WBC), 11,900/μl]; increased platelets (PLT, 480,000/μl); hemoglobin (HgB), 11.5 g/dl; and increased inflammatory indices [C-reactive protein (CRP), 2.5 mg/dl]. She was apyretic.

### 2.3. Therapeutic intervention and histopathological findings

**Figure 3:**
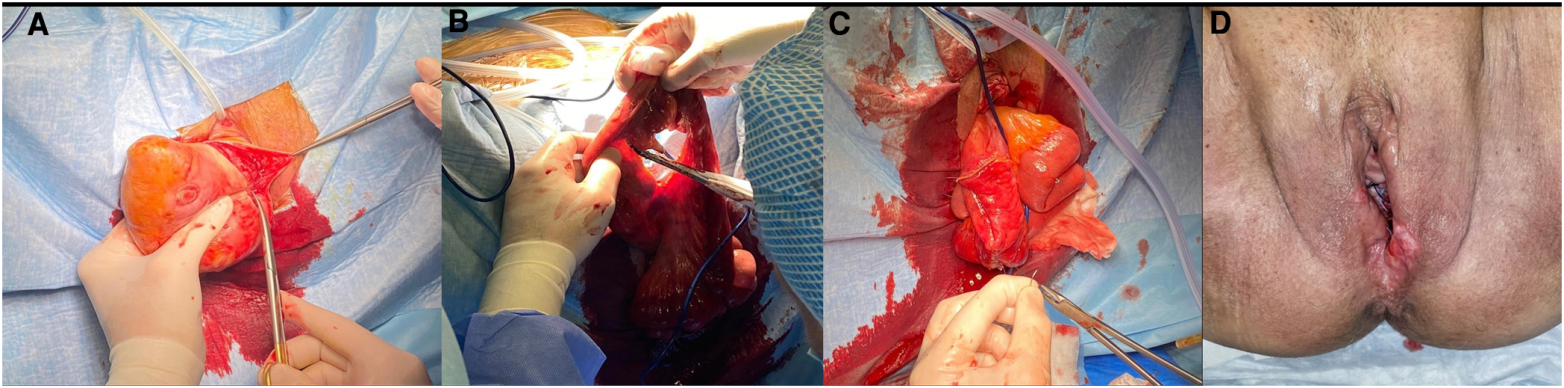
Main steps of the surgical procedure. (**A**) Incision of the vaginal wall with a cold scalpel, (**B**) resection of the small intestine loop, (**C**) subsequent lateral anastomosis with breach sutures, and (**D**) postoperative outcome with a view of the reconstructed vagina.

She was diagnosed with an enterocele with early signs of complications, including initial vaginal rupture, heavy blood loss from ulcerations, and bowel sub-occlusion. Owing to her advanced clinical condition and comorbidities, we opted to perform an urgent vaginal procedure. First, we administered antibiotic prophylaxis. After injection of an ischemic solution into the vaginal wall, a quantity of healthy tissue useful for reconstruction of the vaginal wall was identified and incised using a cold scalpel (Figure 3A). The hernial sac was identified and opened to reveal numerous loops of the small intestine, one of which was visibly edematous and congested, with an initial area of necrosis. Therefore, the aforementioned loop was dissected using Ligasure™ (Medtronic, Minneapolis, MN, USA) and an EndoGIA™ mechanical stapler (Medtronic, Minneapolis, MN, USA), and a lateral anastomosis was subsequently created with breach sutures using a 3-0 V-lock™ (Medtronic, Minneapolis, MN, USA) and 3-0 Vicryl (Ethicon, Raritan, NJ, USA) for the second layer (Figures 3B,C). After checking the intestinal loops, they were carefully repositioned in the abdominal cavity using a large volume of iodine solution. With the patient placed in the Trendelenburg position, the peritoneal layer, muscle, and fascia were sutured continuously with 1-0 Vicryl (Ethicon, Raritan, NJ, USA). We then removed excess vaginal tissue and reconstructed the vagina (Figure 3D) with introflecting stitches using non-absorbable multifilament sutures (Covidien, Mansfield, MA, USA).

**Figure 4:**
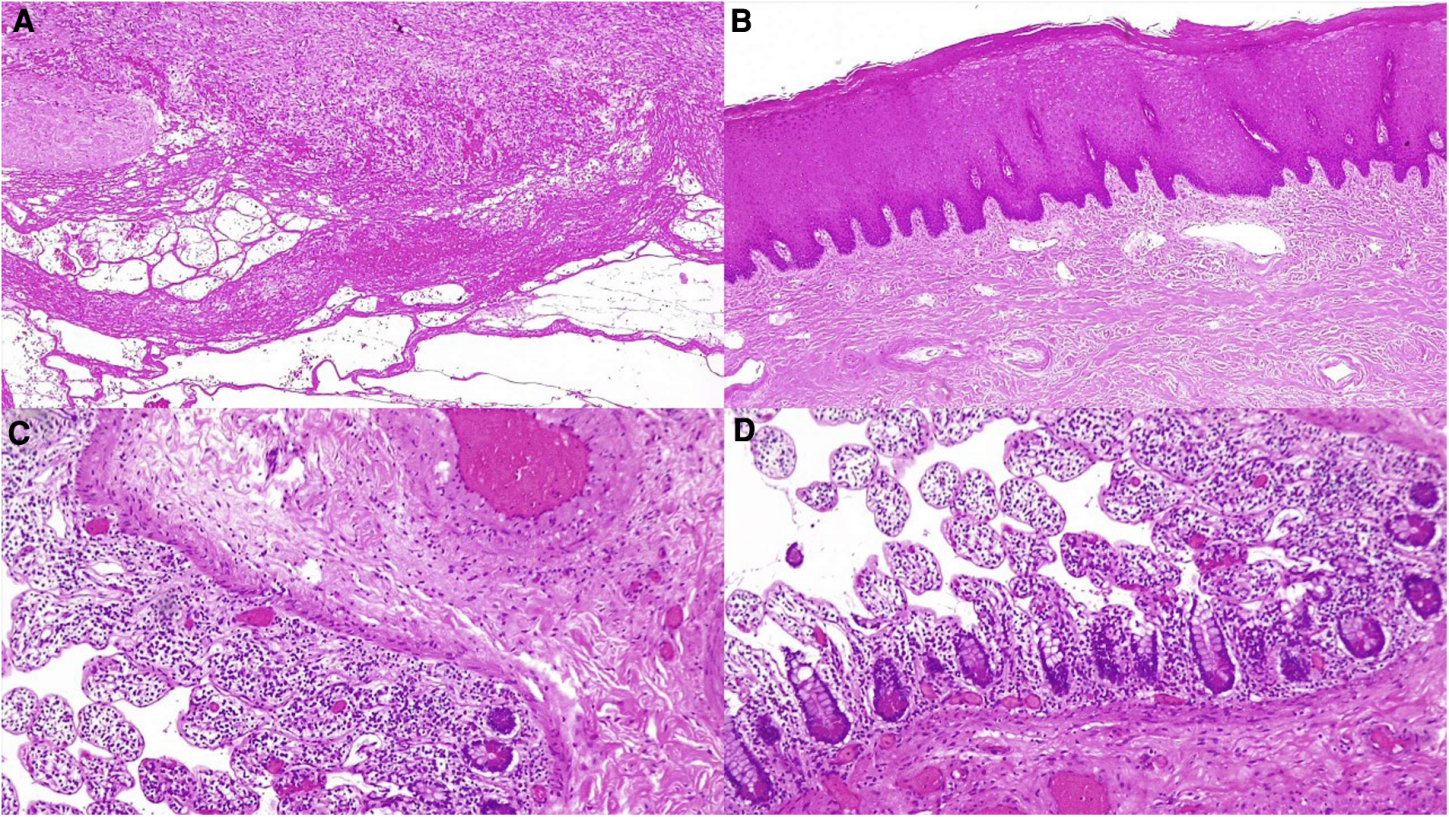
Histological examination of the removed vagina and small bowel (hematoxylin and eosin 4x). (**A**) The vaginal wall with marked ectasia, lymphatic and vascular congestion in the stroma with severe edema, and exacerbated fibro-adherent serositis of the peritoneum lining the prolapsed vagina; (**B**) the vaginal wall lined with multilayered keratinizing squamous epithelium, marked ectasia, and lymphatic and vascular congestion in the stroma; and (**C,D**) small bowel sections showing ischemic-hemorrhagic necrosis of the small bowel mucosa and submucosal layer.

Histological examination showed a vaginal wall lined with multilayered keratinizing squamous epithelium with marked ectasia, lymphatic and vascular congestion in the stroma, severe edema, and exacerbated fibro-adherential serositis of the peritoneum lining the prolapsed vagina. Small bowel sections showed transmural ischemic-hemorrhagic necrosis complicated with ulceration (Figure 4).

### 2.4. Outcomes and follow-up

At 48 h postoperatively, the patient's bowel was channeled with gas. Subsequently, a liquid diet was introduced. Blood examinations were then performed, indicating resolution of inflammation (WBC, 9,800/μl; HgB, 9.9 g/dl; PLT, 341,000/μl; and CRP, 0.5 mg/dl). On postoperative day (POD) 3, the bladder catheter was removed with normal diuresis recovery. On POD 4, the bowel was channeled back to feces, and a regular diet was gradually reintroduced. The patient was discharged on POD 5 in good general and local condition. At a 1-week follow-up, the patient's clinical condition was excellent. At 1 month, she did not show evidence of recurrence or complications. Supplementary Figure S1 illustrates the case report timeline according to the CARE guidelines.

## 3. Discussion

Enterocele can be a dangerous condition that requires accurate and early diagnosis to prevent complications typical of hernias, such as intestinal obstruction, incarceration, and strangulation, with consequent ischemia and necrosis. Most reported cases have presented a clinical situation of vaginal prolapse in which herniated loops are viable and easily reducible without having to undergo resection (12). In 1864, Hypernaux et al. first reported a case of transvaginal prolapse of abdominal content (13).

Transvaginal bowel evisceration, a complication of enterocele, is a rare but serious event. In a review by Kowalsky et al. (14) 63% of patients with vaginal evisceration had enterocele, which had increased stretching of the atrophic vagina; thus, increasing its susceptibility to rupture. Vaginal evisceration is a surgical emergency, and immediate recognition and surgical repair are crucial for its management. Urgent reduction of eviscerated bowel should be undertaken. The surgical approach depends on patient characteristics, bowel viability, and surgeon expertise. Possible approaches include transabdominal, laparoscopic, or transvaginal approaches, or a combination of these (15). In a few cases, when the bowel is not compromised and appears viable, it is easily reducible and there is no evidence of acute abdomen, repair can be accomplished via a vaginal approach. However, in most cases, the bowel cannot be reduced owing to the size of the eviscerated loops and the presence of ischemic outcomes of strangulation, thus requiring laparotomy and transabdominal vaginal repair (11). In particular, in cases where the clinical and laboratory findings suggest an acute abdomen with doubtful peritonitis and in those cases without an adequate preoperative computed tomography imaging, laparotomy with midline subumbilical incision can be recommended to better examine the abdomen. Moreover, in patients with minimal or no enterocele, when the vaginal defect is located high in the vagina, a vaginal repair cannot be pursued because trapped bowel prevents access to the defect and laparotomy is required to reduce and eventually resect the nonviable bowel (9).

Reported cases of pelvic evisceration have mostly involved situations that require intestinal resection due to the presence of one or more necrotic loops. To date, most of these cases have been managed with exploratory laparotomy followed by abdominal or vaginal repair of the defect (9, 11, 16, 17). More recently, cases treated only by laparoscopy (18) or by a combined vaginal laparoscopy approach have been reported (15, 19).

In 2003, Moen et al. reported the first case of vaginal evisceration requiring bowel resection managed via an entirely vaginal approach in an 81-year-old woman (20). In that case, being able to incise the vagina and the very large enterocele sac allowed adequate exposure and sufficient mobility to perform bowel resection and re-anastomosis vaginally, avoiding laparotomy.

In this context, our case differed from most previously described reports as prompt recognition of early signs of necrosis and immediate intervention prevented vaginal bowel evisceration and more severe complications. Moreover, our timely diagnosis enabled us to perform bowel resection and repair surgery via a vaginal approach only.

Currently, no one standard method exists to manage complicated vaginal enterocele or evisceration, with vaginal, laparoscopic, laparotomic, and combined approaches having been reported to be appropriate (10, 15, 21). The surgical approach depends on the clinical circumstances, a surgeon's evaluation, and surgical expertise. Moreover, data are mostly derived from case reports and case series, making it challenging to establish possible risk factors for complications and differences according to surgical procedure type or patient characteristics.

Our experience highlights a rare case of a surgical emergency managed through transvaginal resection of the intestine. This approach requires surgical skill and competency, with the advantage of reduced morbidity and hospitalization rates compared with procedures performed via laparoscopy or laparotomy. A transvaginal approach is less invasive and is associated with reduced postoperative pain, quicker recovery, and shorter length of hospital stay (20). Our patient in particular was able to walk on POD 3 and was discharged on POD 5.

In conclusion, a transvaginal approach, when possible, is preferable even if bowel resection is needed, given the advanced age of patients with this condition. For our patient, this less invasive approach was possible because of the early identification of the initial signs of complications, leading to a favorable patient outcome. Early recognition, as well as timely individualized surgical management, are the cornerstone of reducing morbidity and mortality associated with this subtle and potentially serious condition.

## Data Availability

The raw data supporting the conclusions of this article will be made available by the authors, without undue reservation.

## Supplementary material

Supplementary Figure S1 Case report timeline reported in accordance with CARE guidelines.
