# Supplementary figures and images for: Complicated enterocele: timely resolution with bowel resection via a vaginal approach: case report
Source: Front Surg. 2023 Jul 13;10:1228981. doi: 10.3389/fsurg.2023.1228981 (PMC10372482; doi:10.3389/fsurg.2023.1228981)

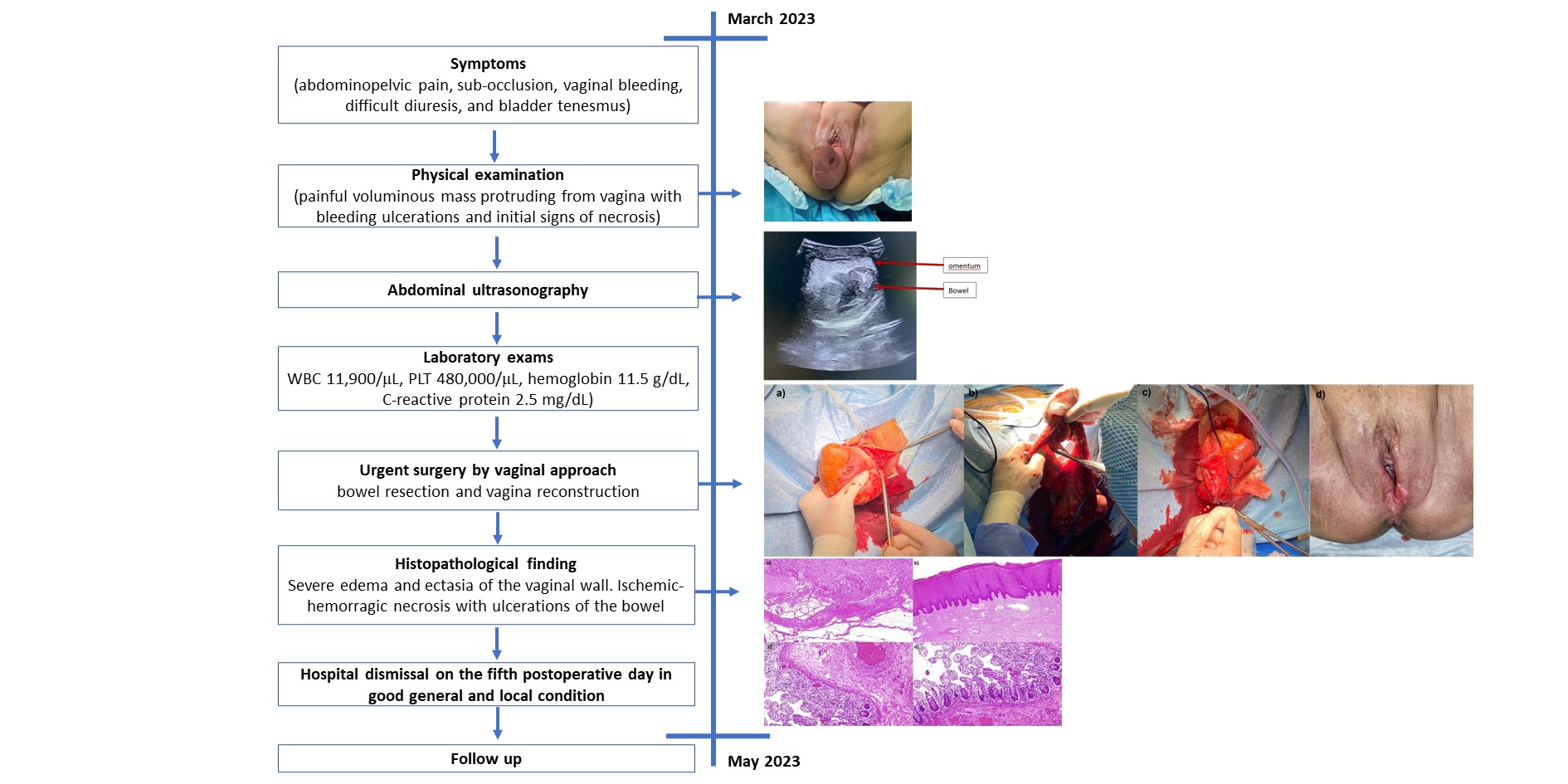

Supplement: Supplementary Figure S1 — Case report timeline reported in accordance with CARE guidelines. [file Image1.jpeg]
